# Supplementary material for: Identifying central elements of the therapeutic alliance in the setting of telerehabilitation: A qualitative study
Source: PLoS One. 2024 Mar 8;19(3):e0299909. doi: 10.1371/journal.pone.0299909 (PMC10923432; doi:10.1371/journal.pone.0299909)
Supplement: S1 Table — (PDF) [file pone.0299909.s002.pdf]

S1 Table. Research team characteristics and attributes.

Author	Characteristics and qualifications
Barbara Seebacher	Female scientist with a PhD degree in physiotherapy including graduate-level training in qualitative research methods, extensive experience with conducting and teaching qualitative research at master's level, and clinical training and advanced practice in neurology, pediatrics, and musculoskeletal rehabilitation
Carole Geimer	Female physiotherapy-scientist studying for a master's degree in therapeutic sciences, with the present research representing a partial fulfilment of her master's degree; MAXQDA and graduate-level training in qualitative research methods, and clinical training and practice as a geriatric physiotherapist
Julia Neu	Female research associate studying for a master's degree in therapeutic sciences, MAXQDA and graduate-level training in qualitative research methods, and clinical training and practice as a musculoskeletal rehabilitation physiotherapist
Maria Schwarz	Female clinical and health psychologist and psychotherapist-scientist with a master's degree, training in cognitive behavioral therapy and communication, and clinical training and advanced practice as a psychologist and psychotherapist in psychosocial rehabilitation and neurology among others

Gudrun Diermayr	Female scientist with a PhD degree in physiotherapy including graduate-level training in qualitative research methods, Professorship at a German university, extensive experience with conducting and teaching qualitative research, and clinical training and advanced practice in neurology
