# Supplementary material for: Identifying central elements of the therapeutic alliance in the setting of telerehabilitation: A qualitative study
Source: PLoS One. 2024 Mar 8;19(3):e0299909. doi: 10.1371/journal.pone.0299909 (PMC10923432; doi:10.1371/journal.pone.0299909)
Supplement: S2 Table — (PDF) [file pone.0299909.s003.pdf]

S2 Table. Keywords used for the literature search and exemplary interview guide.

**Keywords used for the literature search**	
**PubMed and Cochrane Library databases, and Google Scholar search**	Therapeutic alliance AND rehabilitation; therapeutic alliance AND rehabilitation AND (telehealth OR telemedicine OR telerehabilitation); therapeutic (alliance OR relation) AND rehabilitation AND (telehealth OR telemedicine OR telerehabilitation); therapeutic (alliance OR relationship) AND rehabilitation AND (telehealth OR telemedicine OR telerehabilitation) AND working alliance inventory. As a result of the search, an adapted concept map was created, which served as a basis for the design of the interview guidelines.
**Interview guide example of an expert who is a physiotherapist and psychologist**	
**Introduction**	I am pleased that the appointment worked out and that I can interview you today on the topic of therapeutic alliance. First of all, I would like to give you a few explanations. Therapeutic alliance and relationship between therapist and patient are used as synonyms. During the interview I ask you questions about your experience of the therapeutic alliance in physiotherapy and your psychological knowledge. If you did not understand a question, please ask and I will be happy to explain it further.<br>Do you have any further questions before the start?
**Entry questions**	Can you please tell me what TA means to you?

	Which factors of the TA are particularly important to you?
**Questions**	How does communication take place in therapy?<br><br>What channels of communication do you consider relevant?<br><br>Which phases in the process of physiotherapy successful communication is important for?<br><br>In your opinion, does communication influence the relationship between therapist and patient?<br><br>What do you think are the similarities or differences of communication in physiotherapy and psychology / psychotherapy?<br><br>How are goals and tasks set in physiotherapy?<br><br>In your opinion, are there differences between physiotherapy and psychology / psychotherapy?<br><br>Can you please tell me what the roles of patients and therapists should look like?<br><br>Are there possibly other roles besides the patient and therapist roles?<br><br>Tell me, please, what role do you think motivation plays in therapy?<br><br>How can patients be motivated in physiotherapy and in psychology / psychotherapy?<br><br>Are there external factors that you think influence the relationship between therapist and patient? Please explain.<br><br>What influence does time have?

	What influence does safety have?<br><br>What influence does the family have?<br><br>In your opinion, are there similarities or differences between physiotherapy and psychology / psychotherapy?<br><br>Which factors of the TA are particularly important to you?<br><br>What role does trust play in therapy?<br><br>How important is self-disclosure on the part of the therapist and the patient?<br><br>Does empathy play a role in therapy?
	Does humor play a role in therapy?<br><br>What role does professional collaboration play in therapy?<br><br>Is it superior, inferior, or equal to the emotional?<br><br>In your opinion, are there similarities or differences between the TA in physiotherapy and psychology / psychotherapy? Please describe.<br><br>Could you please describe the interaction between the TA and technologies?<br><br>In your opinion, do technologies have an influence on the distribution of roles?<br><br>Does technology impact on the TA in any other way?

**Prompts**	Repetition, paraphrasing, request for clarification or confirmation, such as:<br><br>What else goes through your mind when you hear the term therapeutic alliance?
	Could you tell me something more about …?<br><br>Can you think of anything else to say about …?<br><br>And what else?<br><br>You have mentioned that …. Is there anything you would like to add?<br><br>Could you please elaborate more on that?
