# Supplementary material for: Identifying central elements of the therapeutic alliance in the setting of telerehabilitation: A qualitative study
Source: PLoS One. 2024 Mar 8;19(3):e0299909. doi: 10.1371/journal.pone.0299909 (PMC10923432; doi:10.1371/journal.pone.0299909)
Supplement: S3 Table — (PDF) [file pone.0299909.s004.pdf]

S3 Table. Influencing elements of a successful therapeutic alliance based on the literature search.

Main elements	Secondary elements	Statements	References
**Relationship between the patient and therapist**	Emotional and professional relationship	• The personal, emotional, and professional relationship represent the core components of the therapeutic alliance (TA).<br>• The therapist's empathy and responsiveness play an important role.<br>• Mutual sharing of emotions and self-revelation is a foundation of personal relationship<br>• Common focus between all involved	[1, 2]
		• The patient should be valued with his/her individual physical, psychological, cultural, and social characteristics and needs in relation to personal goals and therapy contents.<br>• The patient is in the foreground, not the disease.	[3-6]
		• For the transmission of knowledge at a professional level	[7]
		• Shared responsibility	[8]

	Roles and responsibilities	• Client-centred approach, which means active participation of the patient.<br>• Defined distribution of roles to avoid conflict	[9, 10]
		• Balance between patient autonomy and therapist support is a challenge	[11]
	Humor	• Humor puts the illness in the background and improves mood.	[1]
	Congruence of the therapist	• Being genuine, open, and authentic during the interactions with the patient is a significant factor of the TA at a professional level	[1]
	Physical presence of a therapist	• Challenge to ensure safety in telerehabilitation	[12]
		• Psychological and physical support by the therapist is relevant and can be guaranteed by his/her presence.	[8, 13]
		• To enable a comprehensive physical examination	[12, 14]
		• Lack of physical presence is a barrier to TA in telerehabilitation	[15]
**Trust**	Aspects of trust	• Exchange of information is important to develop mutual trust.	[3, 16]
		• Positive feedback, empathy and respect promote trust building	[3]

		• A sense of safety strengthens trust	[12]
		• Safety is not directly guaranteed in telerehabilitation and thus a challenge for trust	[17]
**Communi-cation**	Exchange of information	• Exchange of information is essential for the development of TA	[8]
		• Significant for interaction, satisfaction, and therapy success	[18]
	Effective communication	• Effective communication improves the rehabilitation process<br>• Getting to know each other: shared decision-making, congruence between patient and therapist, person-centred communication, respect, active listening, understanding, sympathy and the recognition of the patient as a person with individual needs.<br>• Effective sharing of information, and education of patients: understanding is essential and should be ensured<br>• Joint goal setting and treatment planning is relevant and promoting self-awareness on both sides	[19]
	Communication channels	• Through the body (non-verbal): Facial expression, posture, gestures and / or movements	[20]

		• Verbal and para-verbal: Voice, intonation, flow of speech	[21]
		• General: (1) words and speech, (2) facial expressions and gestures, (3) eyes and gaze, (4) ears and sound of voice, (5) skin and touch, and (6) posture and movement.	[22]
	Communication in telerehabilitation	• In telerehabilitation, non-verbal communication is limited due to the distance	[23]
		• In telerehabilitation, non-verbal communication is limited due to technical aspects	[24]
		• Importance of face-to-face communication for non-verbal aspects of communication	[25]
	Technical aspects	• Time-delay has a negative impact on communication	[25, 26]
**Agreement on tasks and goals**	Agreement on goals	• Agreement between therapist and patient plays an important role in goal setting and decision making in therapy.	[9]
		• Important for the development of TA and thus also for the implementation of therapy	[20]

	Motivation	• Goal setting and consideration of needs increases motivation	[27]
		• The patient's expectation of success and achievement of goals strengthen motivation	[7, 28]
	Autonomy	• Self-management leads to long-term therapy success	[29]
		• Patient engagement is an important aspect especially in telerehabilitation	[30-32]
**External influencing factors**	Relatives and/or caregivers as part of the TA	• Relatives and/or caregivers are part of the TA; it is spoken of as a triad	[33]
		• Relatives and/or caregivers support patients and thus promote the rehabilitation process	[1]
		• Shared relationship between therapist, child and parents	[3]
	Relatives and/or caregivers provide technical support	• Support during the therapy preparation, such as switching on the device or the software programme. • Support during the therapy procedure to position the camera correctly and/or ensure safety.	[10]
		• Clinical environment counteracts development of deep TA	[15, 33]

	Preferred surroundings for developing TA	• Home environment is perceived as a relaxed atmosphere and promotes the building of TA	
		• Some patients want a separation between home and the rehabilitation environment.<br>• Some patients find exercising in the home environment enjoyable.	[15]
	Time as a resource	• Time is a significant factor in building trust.	[34]
		• Time is essential for getting to know each other and for communication.<br>• A sufficient exchange of information makes it easier to take needs into account.	[35]
		• In telerehabilitation, more time is spent with the patient, which promotes TA.	[12]

References for S3 Table

1. Bishop M, Kayes N, McPherson K. Understanding the therapeutic alliance in stroke rehabilitation. Disability and Rehabilitation. 2021;43(8):1074-83. doi: 10.1080/09638288.2019.1651909.
2. Bordin E. The generalizability of the psychoanalytic concept of the working alliance. Psychotherapy. 1979;16:252-60.

3. Crom A, Paap D, Wijma A, Dijkstra PU, Pool G. Between the Lines: A Qualitative Phenomenological Analysis of the Therapeutic Alliance in Pediatric Physical Therapy. Physical & Occupational Therapy In Pediatrics. 2020;40(1):1-14. doi: 10.1080/01942638.2019.1610138.
4. Moore AJ, Holden MA, Foster NE, Jinks C. Therapeutic alliance facilitates adherence to physiotherapy-led exercise and physical activity for older adults with knee pain: a longitudinal qualitative study. J Physiother. 2020;66(1):45-53. Epub 2019/12/18. doi: 10.1016/j.jphys.2019.11.004. PubMed PMID: 31843425.
5. Lawford BJ, Bennell KL, Campbell PK, Kasza J, Hinman RS. Therapeutic Alliance Between Physical Therapists and Patients With Knee Osteoarthritis Consulting Via Telephone: A Longitudinal Study. Arthritis Care & Research. 2020;72(5):652-60. doi: https://doi.org/10.1002/acr.23890.
6. Gómez Penedo JM, Babl AM, Grosse Holtforth M, Hohagen F, Krieger T, Lutz W, et al. The Association of Therapeutic Alliance With Long-Term Outcome in a Guided Internet Intervention for Depression: Secondary Analysis From a Randomized Control Trial. J Med Internet Res. 2020;22(3):e15824. Epub 2020/03/25. doi: 10.2196/15824. PubMed PMID: 32207689; PubMed Central PMCID: PMCPMC7139432.
7. Vestøl I, Debesay J, Pajalic Z, Bergland A. The importance of a good therapeutic alliance in promoting exercise motivation in a group of older Norwegians in the subacute phase of hip fracture; a qualitative study. BMC Geriatrics. 2020;20(1):118. doi: 10.1186/s12877-020-01518-7.
8. Miciak M, Mayan M, Brown C, Joyce AS, Gross DP. A framework for establishing connections in physiotherapy practice. Physiotherapy Theory and Practice. 2019;35(1):40-56. doi: 10.1080/09593985.2018.1434707.
9. Ranner M, Guidetti S, von Koch L, Tham K. Experiences of participating in a client-centred ADL intervention after stroke. Disability and Rehabilitation. 2019;41(25):3025-33. doi: 10.1080/09638288.2018.1483434.
10. Wilms IL. The computerized cognitive training alliance - A proposal for a therapeutic alliance model for home-based computerized cognitive training. Heliyon. 2020;6(1):e03254. Epub 2020/02/12. doi: 10.1016/j.heliyon.2020.e03254. PubMed PMID: 32042977; PubMed Central PMCID: PMCPMC7002830.
11. Cott C. Client-centred rehabilitation: client perspectives. Disability and Rehabilitation. 2004;26(24):1411-22. doi: 10.1080/09638280400000237.
12. Shulver W, Killington M, Morris C, Crotty M. 'Well, if the kids can do it, I can do it': older rehabilitation patients' experiences of telerehabilitation. Health Expectations. 2017;20(1):120-9. doi: https://doi.org/10.1111/hex.12443.
13. Miciak M, Mayan M, Brown C, Joyce AS, Gross DP. The necessary conditions of engagement for the therapeutic relationship in physiotherapy: an interpretive description study. Archives of Physiotherapy. 2018;8(1):3. doi: 10.1186/s40945-018-0044-1.
14. Kairy D, Tousignant M, Leclerc N, Côté A-M, Levasseur M, Researchers TT. The patient's perspective of in-home telerehabilitation physiotherapy services following total knee arthroplasty. International journal of environmental research and public health. 2013;10(9):3998-4011. doi: 10.3390/ijerph10093998. PubMed PMID: 23999548.

15. Cranen K, Drossaert CHC, Brinkman ES, Braakman-Jansen ALM, Ijzerman MJ, Vollenbroek-Hutten MMR. An exploration of chronic pain patients' perceptions of home telerehabilitation services. Health Expectations: An International Journal of Public Participation in Health Care & Health Policy. 2012;15(4):339-50. doi: 10.1111/j.1369-7625.2011.00668.x.
16. Price B. Developing patient rapport, trust and therapeutic relationships. Nurs Stand. 2017;31(50):52-63. Epub 2017/08/10. doi: 10.7748/ns.2017.e10909. PubMed PMID: 28792344.
17. Held JP, Ferrer B, Mainetti R, Steblin A, Hertler B, Moreno-Conde A, et al. Autonomous rehabilitation at stroke patients home for balance and gait: safety, usability and compliance of a virtual reality system. European Journal of Physical and Rehabilitation Medicine 2018;54(4):545-53. Epub 2017/09/28. doi: 10.23736/s1973-9087.17.04802-x. PubMed PMID: 28949120.
18. O'Keeffe M, Cullinane P, Hurley J, Leahy I, Bunzli S, O'Sullivan PB, et al. What Influences Patient-Therapist Interactions in Musculoskeletal Physical Therapy? Qualitative Systematic Review and Meta-Synthesis. Physical Therapy. 2016;96(5):609-22. doi: 10.2522/ptj.20150240.
19. Jesus TS, Silva IL. Toward an evidence-based patient-provider communication in rehabilitation: linking communication elements to better rehabilitation outcomes. Clinical Rehabilitation. 2015;30(4):315-28. doi: 10.1177/0269215515585133.
20. Babatunde F, MacDermid J, MacIntyre N. Characteristics of therapeutic alliance in musculoskeletal physiotherapy and occupational therapy practice: a scoping review of the literature. BMC health services research. 2017;17(1):375. doi: 10.1186/s12913-017-2311-3.
21. Fuchs T. Non-verbale Kommunikation: Phänomenologische, ent-wicklungspsy-chologi-sche und therapeutische Aspekte. Zeitschrift für Klinische Psychologie und Psychotherapie. 2003;51:333-45.
22. Geuter U. Praxis Körperpsychotherapie – 10 Prinzipien der Arbeit im therapeutischen Prozess. Berlin, Deutschland: Springer; 2019.
23. Egolf DB. Nonverbal Communication and Telerehabilitation. In: Kumar S, Cohn ER, editors. Telerehabilitation. Health Informatics. London: Springer; 2013. p. 41–54.
24. Wade SL, Raj SP, Moscato EL, Narad ME. Clinician perspectives delivering telehealth interventions to children/families impacted by pediatric traumatic brain injury. Rehabilitation Psychology. 2019;64(3):298-306. doi: 10.1037/rep0000268.
25. Lie SS, Karlsen B, Graue M, Oftedal B. The influence of an eHealth intervention for adults with type 2 diabetes on the patient–nurse relationship: a qualitative study. Scandinavian Journal of Caring Sciences. 2019;33(3):741-9. doi: https://doi.org/10.1111/scs.12671.
26. Oftedal B, Kolltveit B-CH, Graue M, Zoffmann V, Karlsen B, Thorne S, et al. Reconfiguring clinical communication in the electronic counselling context: The nuances of disruption. Nursing Open. 2019;6(2):393-400. doi: https://doi.org/10.1002/nop2.218.
27. Oyake K, Suzuki M, Otaka Y, Tanaka S. Motivational Strategies for Stroke Rehabilitation: A Descriptive Cross-Sectional Study. Frontiers in Neurology. 2020;11. doi: 10.3389/fneur.2020.00553.

28. Lequerica AH, Kortte K. Therapeutic engagement: a proposed model of engagement in medical rehabilitation. Am J Phys Med Rehabil. 2010;89(5):415-22. Epub 2010/04/22. doi: 10.1097/PHM.0b013e3181d8ceb2. PubMed PMID: 20407308.
29. Dobkin BH. Behavioral self-management strategies for practice and exercise should be included in neurologic rehabilitation trials and care. Current opinion in neurology. 2016;29(6):693-9. doi: 10.1097/wco.0000000000000380. PubMed PMID: 00019052-201612000-00005.
30. Dinesen B, Nielsen G, Andreasen JJ, Spindler H. Integration of Rehabilitation Activities Into Everyday Life Through Telerehabilitation: Qualitative Study of Cardiac Patients and Their Partners. Journal of medical Internet research. 2019;21(4):e13281. Epub 2019/04/16. doi: 10.2196/13281. PubMed PMID: 30985284; PubMed Central PMCID: PMCPMC6487348.
31. Graffigna G, Barello S, Bonanomi A, Menichetti J. The Motivating Function of Healthcare Professional in eHealth and mHealth Interventions for Type 2 Diabetes Patients and the Mediating Role of Patient Engagement. Journal of Diabetes Research. 2016;2016:2974521. doi: 10.1155/2016/2974521.
32. Grünloh C, Myreteg G, Cajander Å, Rexhepi H. "Why Do They Need to Check Me?" Patient Participation Through eHealth and the Doctor-Patient Relationship: Qualitative Study. Journal of medical Internet research. 2018;20(1):e11. Epub 2018/01/18. doi: 10.2196/jmir.8444. PubMed PMID: 29335237; PubMed Central PMCID: PMCPMC5789160.
33. Lawton M, Sage K, Haddock G, Conroy P, Serrant L. Speech and language therapists' perspectives of therapeutic alliance construction and maintenance in aphasia rehabilitation post-stroke. International Journal of Language & Communication Disorders. 2018;53(3):550-63. doi: 10.1111/1460-6984.12368.
34. Wilson S, Chaloner N, Osborn M, Gauntlett-Gilbert J. Psychologically informed physiotherapy for chronic pain: patient experiences of treatment and therapeutic process. Physiotherapy. 2017;103(1):98-105. doi: 10.1016/j.physio.2015.11.005.
35. Gard G. Factors important for good interaction in physiotherapy treatment of persons who have undergone torture: A qualitative study. Physiotherapy Theory and Practice. 2007;23(1):47-55. doi: 10.1080/09593980701209584.
