# Supplementary material for: Identifying central elements of the therapeutic alliance in the setting of telerehabilitation: A qualitative study
Source: PLoS One. 2024 Mar 8;19(3):e0299909. doi: 10.1371/journal.pone.0299909 (PMC10923432; doi:10.1371/journal.pone.0299909)
Supplement: S4 Table — (PDF) [file pone.0299909.s005.pdf]

S4 Table. Codes derived from expert interview data including descriptors.

Codes	Subcodes	Descriptors
**Communication**	Effective communication	Effective communication necessary to build the bond
		Communication on an equal basis
		Using the most effective communication channels (verbal, non-verbal, paraverbal) as a therapist
		Ensuring good video quality / lighting to discern a change in facial expression and gestures in telerehabilitation
		Exchanging information in a timely manner
	Clear instructions	Providing the patient with unambiguous instructions, particularly in telerehabilitation where tactile facilitation is not possible
	Mutual feedback	Mutual feedback using the most appropriate communication channels
	Touch	Using touch as a means to treat and communicate in rehabilitation
		Advising caregivers and the family on how to use professional touch in telerehabilitation
		Caregivers and relatives acting as co-therapists using professional touch

	Advising on safety	Advising caregivers and family on how to safeguard the patient in telerehabilitation
	Appropriate means of communication	Choosing the most appropriate means of communication in rehabilitation (personal, written, video)
		Choosing the most appropriate means of communication in telerehabilitation (time-delayed, real-time, email, video)
**Bond**	Safeguarding	Safeguarding the patient as a prerequisite for building trust
	Presence	Being present as a therapist
		Being present as a patient
	Responsiveness	Being responsive as a therapist
	Openness	Being open for the physiotherapist's suggestions as a patient
	Self-disclosure	Self-disclosure and openness of the therapist and patient to strengthen their bond
	Genuinely caring	Being genuinely caring as a therapist
	Respect	Respecting each other
	Acceptance	Accepting each other
	Honesty	Being honest
	Friendly interactions	The therapist being friendly and warm in their interactions with the patient

		The patient being friendly and sociable in their interactions with the therapist
	Self-reflexion	Being self-reflective as a therapist
	Congruence and authenticity	Congruence and authenticity of the therapist
	Humour	Humour to intensify the therapeutic relationship
	Appreciation	Appreciating the patient as a person
	Building an emotional relationship	Emotional relationship between the patient and therapist
		Acknowledging and validating the patient's fears, expectations and hopes
		Acknowledging the patient's emotional responses
		Acknowledging the influence of technology on the patient's emotional responses in telerehabilitation
		Recognising the potential influence of technology on the therapeutic relationship
	Building a professional relationship	Defining the therapist's and patient's roles
		Appreciating the therapist's professional competence and guidance
		Recognising the patient's emotional, cognitive and physical needs

		Understanding the patient's needs and fears
		Responding to the patient's needs
		Delivering comprehensive information for the patient
		Strengthening the patient's awareness of his / her clinical problem
		The patient being receptive for the therapist's advice
		Active listening of the therapist and patient
		Shared decision-making
		Patient education
**Trust**	Safety	Reducing risks and enhancing safety, respectively, particularly in telerehabilitation to increase trust of the patient
	Respect	Building mutual trust through transparent and respectful communication
	Transparency	
	Empathy	Being empathetic as a therapist as a prerequisite for building trust
	Mutual exchange	Enhancing trust through mutual exchange
	Continuous care	Strengthening trust of the patient through continuous (tele-)rehabilitation care

**Agreeing on goals and tasks**	Similarity of goals	Delivering comprehensive information for the patient
	Shared responsibility	Strengthening the patient's awareness of his / her clinical problem
		Shared responsibility for a successful task completion
	Goal setting	The patient being receptive for the therapist's advice
	Defining tasks	Active listening of the therapist and patient
	Targeting	Shared decision-making
	Individualising	Patient education
	Encouraging	Encouraging the patient
	Adherence	Patient's adherence with performing tasks
	Congruence	Congruence between the patient and therapist on goal setting in (tele-)rehabilitation
**Patient autonomy and self-management**	Being prepared	The patient's need to prepare him-/herself for using telerehabilitation
		Start of telerehabilitation session only once the therapist and patient are ready
	Self-efficacy	Facilitation of self-efficacy of the patient in telerehabilitation
	Self-management	Greater need for the patient's self-management in telerehabilitation

	Autonomy	Higher autonomy of the patient in telerehabilitation necessary
**Motivation of the patient**	Motivating factors in rehabilitation and telerehabilitation	Being heard by the therapist
		Receiving therapist's response to one's individual questions
		The therapist approving the patient's efforts
		Being encouraged by the therapist
		Receiving direct (real-time) feedback from the therapist
		Receiving direct (real-time) feedback from the patient
		Working on jointly defined therapy goals
		Experiencing improvement of motor function through (tele-)rehabilitation
		Virtual reminders
		Virtual reward systems
		Virtual display of success in therapy
	Further motivating factors	Being able to access telerehabilitation services despite COVID-19
**Agreeing on roles**	Roles of the therapist	The therapist acting as a technical supporter
		The therapist acting as a rehabilitation expert

		The therapist acting as an expert despite a virtual surrounding
	Roles of the patient	With respect to the role(s) of the patient, individual needs to be accounted for by the therapist
		The patient at the centre of the therapy
		A clear distribution of roles between the therapist and patient to enhance the therapeutic relationship
	Roles of the family and caregivers	Ensuring the patient's physical safety in telerehabilitation
		As co-therapist in telerehabilitation
		Supporting goal setting in (tele)rehabilitation
		Addressing the patient's needs in rehabilitation (travel to the clinic, physical support etc.)
		Coordinating / supporting telerehabilitation preparations (material, technological)
		Being the extended arm of the therapist in telerehabilitation
		Helping in task performance of the patient in telerehabilitation

		Selecting tasks / supporting unsupervised homebased practice of the patient in (tele)rehabilitation
		Supporting the patient with their use of technology in telerehabilitation
**External factors**	Home environment	Patient's home environment (in contrast to a rehabilitation clinic environment)
		Risks (of falls) due to the absence of the therapist
		Preparation and follow-up times need to be considered for a telerehabilitation setting
	Time	Time for the therapy in (tele)rehabilitation
		Time required for technical problem solving of the therapist/caregivers in telerehabilitation
		The amount of time corresponding to the efforts of the therapist and caregivers or the patient
	Supporters	The family and caregivers
	Technology-related aspects to consider in telerehabilitation	Virtual connection impacting on the patient's emotional state
		Virtual connection impacting on the patient's trust in the therapist
		Virtual connection impacting on the bond between the therapist and patient

		Taking precautions to ensure safety in telerehabilitation
		Data protection
		Material costs / requirements
		(Dys-)function of telerehabilitation programmes
		Challenges related to using technology in some patient groups
	Potentially disruptive factors in telerehabilitation	Failure of the WiFi connection
		Inability to meet technical requirements
		Technological failure
		Using unfamiliar telerehabilitation software and/or hardware as a therapist
		Introducing telerehabilitation software and/or hardware unfamiliar to the patient
		Failure of real-time data transmission
		Therapist's or patient's hesitancy towards using technology or telerehabilitation
		Data backup failure in telerehabilitation
**Differences between telerehabilitation and**	Preparation time	A longer preparation time in telerehabilitation except when using ready-to-use programmes
	Active role of the patient	The patient being placed in a more active role in telerehabilitation.

**conventional therapy**	Change in interaction	In telerehabilitation, potential shift of the interaction with the patient towards using telerehabilitation programmes / games.
	Hands-on	In telerehabilitation, hands-on techniques or professional touch not possible.
	Field of action	In telerehabilitation, potentially smaller field of action compared to conventional rehabilitation.
